# A Nomogram for Predicting Cancer-Specific Survival of Osteosarcoma and Ewing's Sarcoma in Children: A SEER Database Analysis

**DOI:** 10.3389/fpubh.2022.837506

**Published:** 2022-02-01

**Authors:** Jinkui Wang, Chenghao Zhanghuang, Xiaojun Tan, Tao Mi, Jiayan Liu, Liming Jin, Mujie Li, Zhaoxia Zhang, Dawei He

**Affiliations:** ^1^Department of Urology, Chongqing Key Laboratory of Children Urogenital Development and Tissue Engineering, Chongqing Key Laboratory of Pediatrics, Ministry of Education Key Laboratory of Child Development and Disorders, National Clinical Research Center for Child Health and Disorders, China International Science and Technology Cooperation Base of Child Development and Critical Disorders, Children's Hospital of Chongqing Medical University, Chongqing, China; ^2^Department of Urology, Kunming Children's Hospital (Children's Hospital Affiliated to Kunming Medical University), Yunnan Key Laboratory of Children's Major Disease Research, Kunming, China; ^3^Department of Urology, Nanchong Central Hospital, the Second Clinical Medical College, North Sichuan Medical University, Nanchong, China

**Keywords:** nomogram, cancer-specific survival, Osteosarcoma, Ewing's sarcoma, children

## Abstract

**Background:**

Osteosarcoma (OSC) and Ewing's sarcoma (EWS) are children's most common primary bone tumors. The purpose of the study is to develop and validate a new nomogram to predict the cancer-specific survival (CSS) of childhood OSC and EWS.

**Methods:**

The clinicopathological information of all children with OSC and EWS from 2004 to 2018 was downloaded from the Surveillance, Epidemiology, and End Results (SEER) database. Univariate and multivariate Cox regression analyses were used to screen children's independent risk factors for CSS. These risk factors were used to construct a nomogram to predict the CSS of children with OSC and EWS. A series of validation methods, including calibration plots, consistency index (C-index), and area under the receiver operating characteristic curve (AUC), were used to validate the accuracy and reliability of the prediction model. Decision curve analysis (DCA) was used to validate the clinical application efficacy of predictive models. All patients were divided into low- and high-risk groups based on the nomogram score. Kaplan-Meier curve and log-rank test were used to compare survival differences between the two groups.

**Results:**

A total of 2059 children with OSC and EWS were included. All patients were randomly divided into training cohort 60% (*N* = 1215) and validation cohort 40% (*N* = 844). Univariate and multivariate analysis suggested that age, surgery, stage, primary site, tumor size, and histological type were independent risk factors. Nomograms were established based on these factors to predict 3-, 5-, and 8-years CSS of children with OSC and EWS. The calibration plots showed that the predicted value was highly consistent with the actual value. In the training cohort and validation cohort, the C-index was 0.729 (0.702–0.756) and 0.735 (0.702–0.768), respectively. The AUC of the training cohort and the validation cohort also showed similar results. The DCA showed that the nomogram had good clinical value.

**Conclusion:**

We constructed a new nomogram to predict the CSS of OSC and EWS in children. This predictive model has good accuracy and reliability and can help doctors and patients develop clinical strategies.

## Introduction

Osteosarcoma (OSC) and Ewing sarcoma (EWS) are the most common primary malignant bone tumors in children and young people (1–3). OSC is the most common tumor in children and adolescence, and its peak incidence occurs in adolescence. The annual incidence rate for children under 10 years old is 1.7 per 100,000, while the yearly incidence rate for patients between 10 and 19 is 8.2 per 100,000 (4). Although various treatments are used to improve the prognosis, the survival rate of OSC is still shallow (5–7), the 5-year survival rate of patients with localized OSC can reach 65–70% (8). In comparison, the survival rate of patients with metastatic OSC is only 19–30% (9, 10). EWS is a malignant bone tumor commonly seen in children and adolescents. Its incidence is second only to OSC, accounting for 3% of solid malignant tumors in children (2, 3). In recent years, with the improvement of treatment technology, the 5-year survival rate of EWS has reached 60% to 70%, but the prognosis of patients with metastasis and recurrence is still poor (11).

Yang et al. (12) established a nomogram to predict the cancer-specific survival (CSS) and overall survival rate of OSC. It showed that age, stage, grade, surgery, primary site, and tumor size were independent risk factors for OSC. Lu et al. (13) established a nomogram to predict the risk of distant metastasis of OSC. We found that age, tumor location, tumor grade, T stage, and surgical method were risk factors for metastasis and suggested a worse prognosis. Zhou et al. (14) established a nomogram to predict the overall survival of EWS, revealing that age, N stage and bone metastasis are independent risk factors for prognosis. However, as far as we know, no nomogram has been established for children with OSC and EWS to predict tumor-specific survival.

At present, artificial intelligence has been widely used in human health. Dhanamjayulu et al. (15) used machine learning to identify malnourished people. Gadekallu et al. (16) used a neural network to predict retinopathy in diabetic patients. Reddy et al. (17) used an artificial intelligence algorithm to diagnose heart disease. Abbas et al. (18) used a new algorithm to monitor breast cancer. Mubashar et al. (19) use artificial intelligence to manage personal health records.

The nomogram is a way to estimate the occurrence of a given event by generating corresponding values from the clinical-pathological information of the patient. At present, nomograms have been widely used in the clinical prediction of liver cancer, lung cancer, and breast cancer (20–22). As the two most common bone malignancies in children, OSC and EWS seriously endanger children's health. Revealing the prognostic factors of childhood OSC and EWS can help doctors and patients choose appropriate treatment measures, which is conducive to prolonging the survival time of patients and improving the quality of life. In other words, accurately predicting the prognosis of malignant tumors can help patients and doctors formulate treatment plans and follow-up strategies.

However, the prediction models for OSC and EWS in children are still not well established. No studies have precisely predicted CSS in pediatric OSC and EWS patients. Bone tumor in children is an essential factor endangering children's health. Accurate prediction of the survival of bone tumors in children can enhance the population's health and improve people's overall quality of life and life span. Therefore, it is necessary to construct a nomogram to predict the survival prognosis of childhood OSC and EWS.

## Patients and Methods

### Data Source and Data Extraction

Patient data are downloaded from the Surveillance, Epidemiology, and End Results (SEER) project of the National Cancer Institute. From 2004 to 2018, all children under 18 diagnosed with OSC and EWS were collected. The SEER database is a cancer database in the United States, covering about 28% of Americans, and contains 18 cancer registries (23). The SEER database discloses patient demographic information, clinicopathological characteristics, and survival status. The clinicopathological information we used is public and anonymous, so our study does not require ethical approval and patient consent. Our study method complies with the regulations of the SEER database.

**Figure 1:**
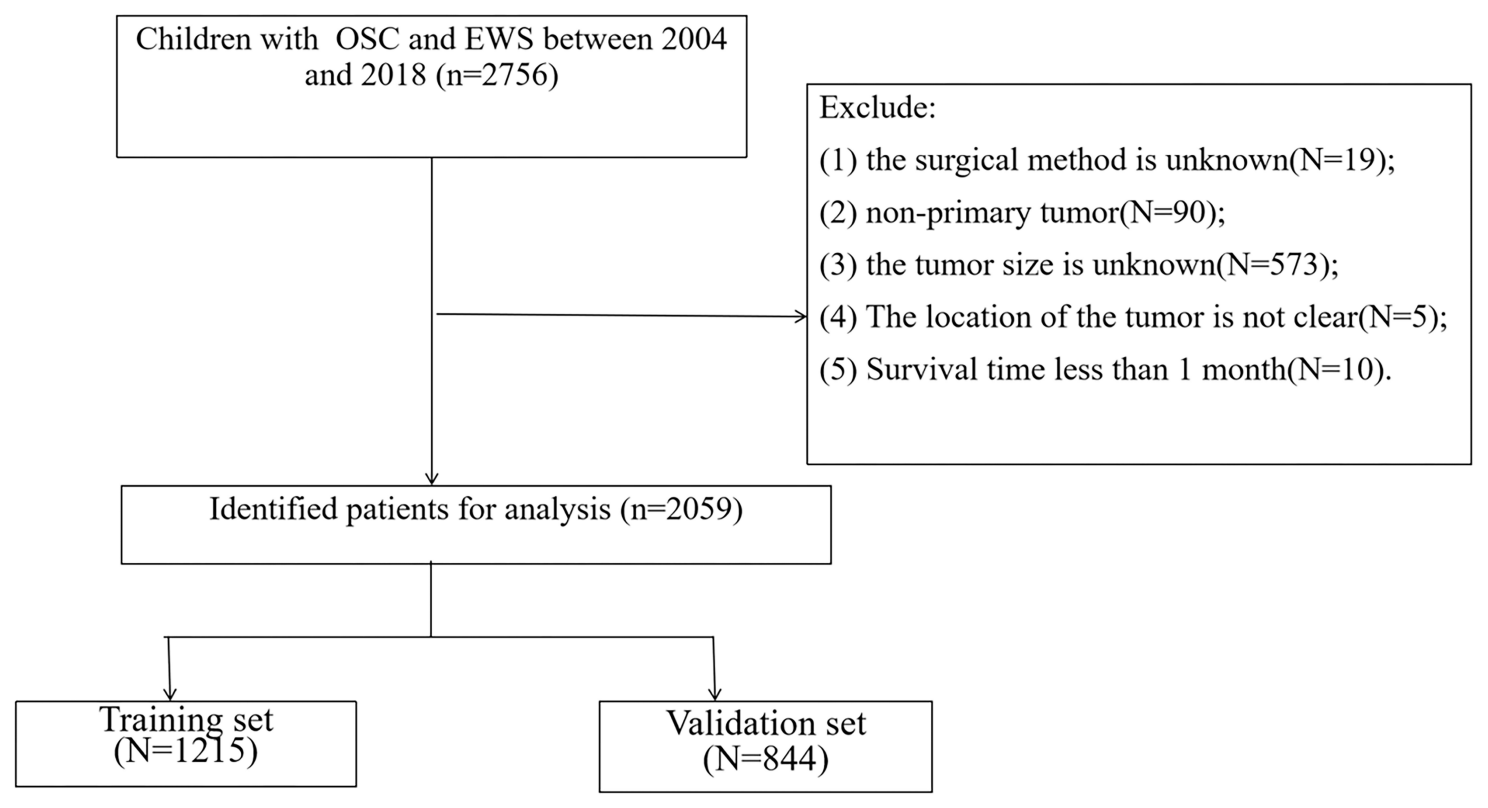
The flowchart of including and dividing patients.

We collected patient demographic information (age, sex, race), tumor characteristics (tumor grade, size, primary location, pathological type), treatment (surgery, radiotherapy, chemotherapy), follow-up information (survival status, survival time). The selection criteria are: (1) age less than 18 years old; (2) diagnosed as OSC and EWS. The exclusion criteria are: (1) the surgical method is unknown; (2) non-primary tumor; (3) the tumor size is unknown; (4) The location of the tumor is not precise; (5) Survival time less than 1 month. The flowchart of patient screening is shown in Figure 1.

The race of all children was divided into white, black, and other (American Indian/AK Indian, Asian/Pacific Islander). The tumor grade was split into I (well-differentiated), II (medium differentiated), III (poorly differentiated), IV (undifferentiated). According to the SEER surgery code, the surgery methods were divided into four groups: no surgery, partial resection, radical excision, and amputation. The primary site of the tumor was divided into the limb, cranial, spine, thoracic, and pelvic.

### Univariate and Multivariate Cox Regression Analysis

All patients were randomly divided into a training set (60%) and a validation set (40%). Univariate Cox regression screened out related prognostic factors. Then multivariate Cox regression analysis was used to analyse the independent risk factors of the training set. And we recorded the hazard ratio (HR) and 95% confidence interval (CI) simultaneously.

### Nomogram Construction

Based on the independent risk factors obtained by univariate and multivariate Cox regression, a nomogram was constructed to predict the 3-, 5-, 8-year CSS of children with OSC and EWS. Each variable is distributed on the nomogram according to its weight to get different lines, and the points of each variable correspond to a point. The sum of the points of all the variables in the nomogram can equal an overall points, thus obtaining survival rates at different points in time.

### Nomogram Validation

Then a series of validation methods were used, including the consistency index (C-index), the calibration curve, and the area under the receiver operating characteristic curve (AUC). The accuracy of the nomogram was tested mainly by the calibration curve, which was used to compare the relationship between the observed value and the actual value through 1,000 bootstrap sampling. The results of the calibration curve are dotted in the **Figure 3**. If they are close to the diagonal, the model has good accuracy. Both the C-index and the AUC are used to assess the discrimination of the model.

### Clinical Utility

Decision curve analysis (DCA) is a new algorithm to evaluate the clinical value of the model based on the net benefit under each risk threshold (24). DCA was used to assess the clinical potential application value of the new nomogram. Subsequently, we divided the patients into a low-risk group and a high-risk group through the nomogram score of each patient. The Kaplan-Meier curve and log-rank test were used to compare the survival differences of different risk groups. At the same time, we analyzed the surgical methods of patients in other risk groups.

### Statistical Analysis

All statistics were performed using SPSS23.0 (IBM, Chicago, IL, USA) and R software (version 3.4.1; http://www.Rproject.org). Age and tumor size were continuous variables and did not follow a normal distribution; median and the inter-quartile range was used for description. All possible prognostic factors were confirmed using univariate Cox regression. Then, the relevant variables were included in the multivariate analysis and the stepwise regression method was used to select the best Cox regression model. A *p* < 0.05 was considered statistically significant.

## Results

### Clinical Features

A total of 2,059 children with OSC and EWS were included in our study. They were divided into a training set (*N* = 1215) and a validation set (*N* = 844). The clinicopathological characteristics are shown in Table 1. There was no significant difference between the training set and the validation set. The median age of all children was 13 years old (interquartile range, 10–15 years old), including 1,179 males (57.3%) and 1,624 whites (78.9%). One thousand three hundred eighty-five cases (67.3%) were OSC, and 674 cases (32.7%) were EWS. Children whose primary tumors were located in limb, cranial, spine, thoracic and pelvic were 1,576 cases (76.5%), 93 cases (4.52%), 56 cases (2.72%), 118 cases (5.73%), and 216 cases (10.5%), respectively. Children with tumor grades I, II, III, and IV was 23 (1.12%), 48 (2.33%), 419 (20.3%), and 747 (36.3%), respectively. There were 673 cases (32.7%), 842 cases (40.9%), and 531 cases (25.8%) of children whose tumor stages were localized, regional and distant, respectively. The median tumor size was 90 mm (interquartile range, 62–125 mm). Most children have performed chemotherapy (1,978, 96.1%), and most have not completed radiotherapy (1,674, 81.3%). Partial resection, radical excision and amputation were performed in 230 cases (11.2%), 1,166 cases (56.6%), and 342 cases (16.6%). A total of 474 patients (23.0%) had distant metastases.

**Table 1:** Clinicopathological characteristics of children with bone tumor.

	**Total (*N* = 2,059)**	**Training cohort (*N* = 1,215)**	**Validation cohort (N = 844)**	***p***
Age (median [IQR])	13 (10, 15)	13 (10, 15)	13 (10, 15)	0.6892
Race				0.717
White	1,624 (78.9%)	955 (78.6%)	669 (79.3%)	
Black	233 (11.3%)	143 (11.8%)	90 (10.7%)	
Other	202 (9.81%)	117 (9.63%)	85 (10.1%)	
Sex				0.020
Male	1,179 (57.3%)	722 (59.4%)	457 (54.1%)	
Female	880 (42.7%)	493 (40.6%)	387 (45.9%)	
Grade				0.933
I	23 (1.12%)	14 (1.15%)	9 (1.07%)	
II	48 (2.33%)	27 (2.22%)	21 (2.49%)	
III	419 (20.3%)	248 (20.4%)	171 (20.3%)	
IV	747 (36.3%)	433 (35.6%)	314 (37.2%)	
Unknown	822 (39.9%)	493 (40.6%)	329 (39.0%)	
Stage				0.591
Localized	673 (32.7%)	398 (32.8%)	275 (32.6%)	
Regional	842 (40.9%)	484 (39.8%)	358 (42.4%)	
Distant	531 (25.8%)	325 (26.7%)	206 (24.4%)	
Unstaged	13 (0.63%)	8 (0.66%)	5 (0.59%)	
Year of diagnosis				0.651
2004–2010	860 (41.8%)	502 (41.3%)	358 (42.4%)	
2010–2018	1,199 (58.2%)	713 (58.7%)	486 (57.6%)	
Primary site				0.713
Limb	1,576 (76.5%)	926 (76.2%)	650 (77.0%)	
Cranial	93 (4.52%)	55 (4.53%)	38 (4.50%)	
Spine	56 (2.72%)	35 (2.88%)	21 (2.49%)	
Thoracic	118 (5.73%)	76 (6.26%)	42 (4.98%)	
Pelvic	216 (10.5%)	123 (10.1%)	93 (11.0%)	
Histologic type				0.832
Osteogenic sarcoma	1,385 (67.3%)	820 (67.5%)	565 (66.9%)	
Ewings sarcoma	674 (32.7%)	395 (32.5%)	279 (33.1%)	
Laterality				0.789
Left	952 (46.2%)	563 (46.3%)	389 (46.1%)	
Right	901 (43.8%)	535 (44.0%)	366 (43.4%)	
Not a paired site	206 (10.0%)	117 (9.63%)	89 (10.5%)	
Chemotherapy				0.189
No/Unknown	81 (3.93%)	54 (4.44%)	27 (3.20%)	
Yes	1,978 (96.1%)	1,161 (95.6%)	817 (96.8%)	
Radiation				0.937
No/Unknown	1,674 (81.3%)	989 (81.4%)	685 (81.2%)	
Yes	385 (18.7%)	226 (18.6%)	159 (18.8%)	
Surgery				0.926
No	321 (15.6%)	193 (15.9%)	128 (15.2%)	
Partial resection	230 (11.2%)	136 (11.2%)	94 (11.1%)	
Radical excision	1,166 (56.6%)	681 (56.0%)	485 (57.5%)	
Amputation	342 (16.6%)	205 (16.9%)	137 (16.2%)	
Tumor size (median [IQR])	90 (62, 125)	90 (63, 125)	88.5 (61.75, 125.25)	0.7592
Metastasis				0.469
No/Unknown	1,585 (77.0%)	928 (76.4%)	657 (77.8%)	
Yes	474 (23.0%)	287 (23.6%)	187 (22.2%)	

### Univariate and Multivariate Cox Regression Analysis

We first established a univariate Cox regression model to screen possible prognostic factors. Then we established a Cox multivariate model to identify independent risk factors affecting OS in children. Univariate and multivariate Cox regression results are shown in Table 2. Finally, we found age (HR 1.030; 95% CI 1.006–1.055), tumor size (HR 1.002; 95% CI 1.001–1.003), histological type (HR 0.563; 95% CI 0.434–0.73), surgery, stage, and primary site were independent risk factors. In other words, these six factors can be used to establish a nomogram to predict the CSS of OSC and EWS in children.

**Table 2:** Univariate and multivariate analyses of CSS in bone tumor.

	**Univariate**			**Multivariate**		
	**HR**	**95% CI**	***p***	**HR**	**95% CI**	***p***
Age	1.06	1.03–1.09	<0.0001	1.030	1.006–1.055	0.015
Sex						
Male	Reference					
Female	0.87	0.72–1.07	0.19			
Year of diagnosis						
2004–2010	Reference					
2011–2018	0.53	0.43–0.64	<0.0001			
Race						
White	Reference					
Black	1.13	0.83–1.53	0.45			
Other^a^	0.94	0.67–1.31	0.71			
Primary site						
Limb	Reference					
Cranial	0.45	0.24–0.83	0.01	0.772	0.425-1.401	0.394
Spine	1.21	0.67–2.19	0.52	1.760	1.021–3.033	0.042
Thoracic	1.03	0.67–1.59	0.88	1.232	0.814–1.864	0.324
Pelvic	2.12	1.58–2.85	<0.0001	1.396	1.045–1.863	0.024
Grade						
I	Reference					
II	0.48	0.14–1.65	0.25			
III	0.85	0.33–2.22	0.75			
IV	1.17	0.45–3	0.75			
Unknown	0.97	0.38–2.5	0.95			
Histologic type						
Osteogenic sarcoma	Reference			Reference		
Ewings sarcoma	0.89	0.72–1.1	0.28	0.563	0.434–0.73	<0.0001
Laterality						
Left	Reference					
Right	0.91	0.74–1.11	0.35			
Not a paired site	0.99	0.7–1.38	0.93			
Stage						
Localized	Reference			Reference		
Regional	2.3	1.74–3.03	<0.0001	1.743	1.348–2.255	<0.0001
Distant	5.96	4.48–7.93	<0.0001	4.400	3.396–5.701	<0.0001
Unstaged	1.27	0.28–5.85	0.75	1.309	0.319–5.377	0.709
Surgery						
No	Reference			Reference		
Partial resection	0.55	0.37–0.8	<0.0001	0.707	0.499–1.002	0.051
Radical excision	0.46	0.35–0.6	<0.0001	0.503	0.382–0.663	<0.0001
Amputation	0.87	0.63–1.2	0.40	0.742	0.545–1.01	0.058
Radiation						
No/Unknown	Reference					
Yes	1.63	1.28–2.07	<0.0001			
Chemotherapy						
No/Unknown	Reference					
Yes	2.31	1.21–4.4	0.01			
Tumor size	1	1–1.01	<0.0001	1.002	1.001–1.003	0.006
Metastasis						
No	Reference					
Yes	3.58	2.88–4.46	3.58			

a *Other: American Indian/AK Native, Asian/Pacific Islander*.

### Nomogram Construction for 3-Year, 5-Year, and 8-Year CCS

**Figure 2:**
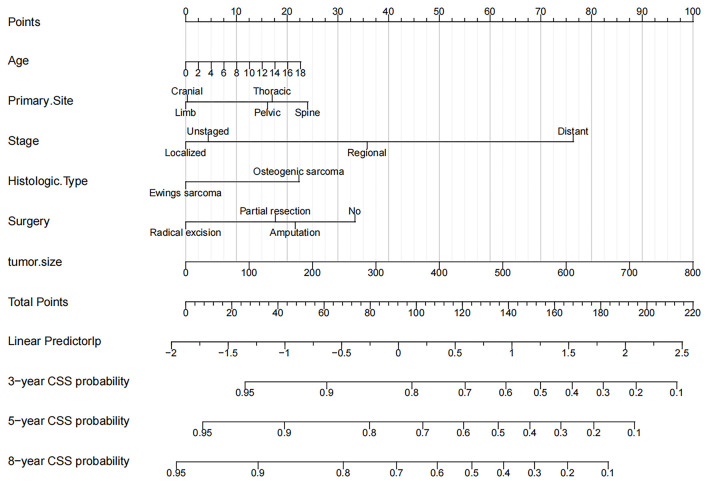
Nomogram for 3-, 5-, and 8-year CSS of children with OSC and EWS.

The six independent risk factors above were used to construct a nomogram to predict CSS in children with OSC and EWS (Figure 2). The nomogram accurately listed the impact of each factor on CSS. We found that tumor size was the most significant influencing factor, and a large tumor meant a higher risk of death. The second was the staging of tumors. There was no doubt that distantly metastatic tumors had a higher risk of death than in localized or regional tumors. The location of the primary tumor was also a significant risk factor. We found that tumors in the spine, pelvis, and thorax have a significantly higher risk of death than tumors in the limbs and skull. In addition, the children with radical resection of the tumor, the younger children and the children with EWS had the lowest mortality.

### Validation of the Nomogram

**Figure 3:**
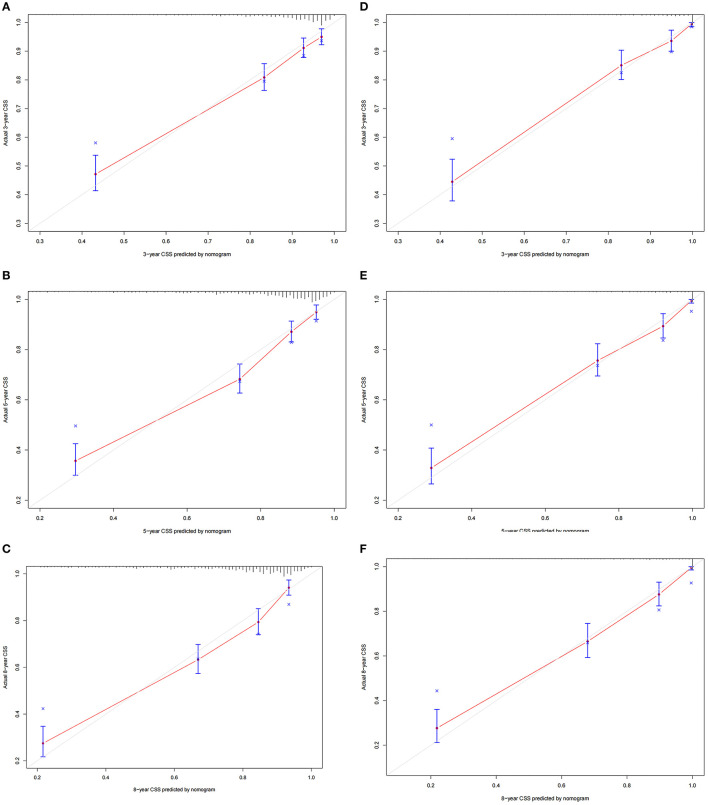
Calibration curve of the nomogram. **(A–C)** For 3-, 5-, and 8-year CSS in the training set; **(D–F)** for 3-, 5-, and 8-year in the validation set.

**Figure 4:**
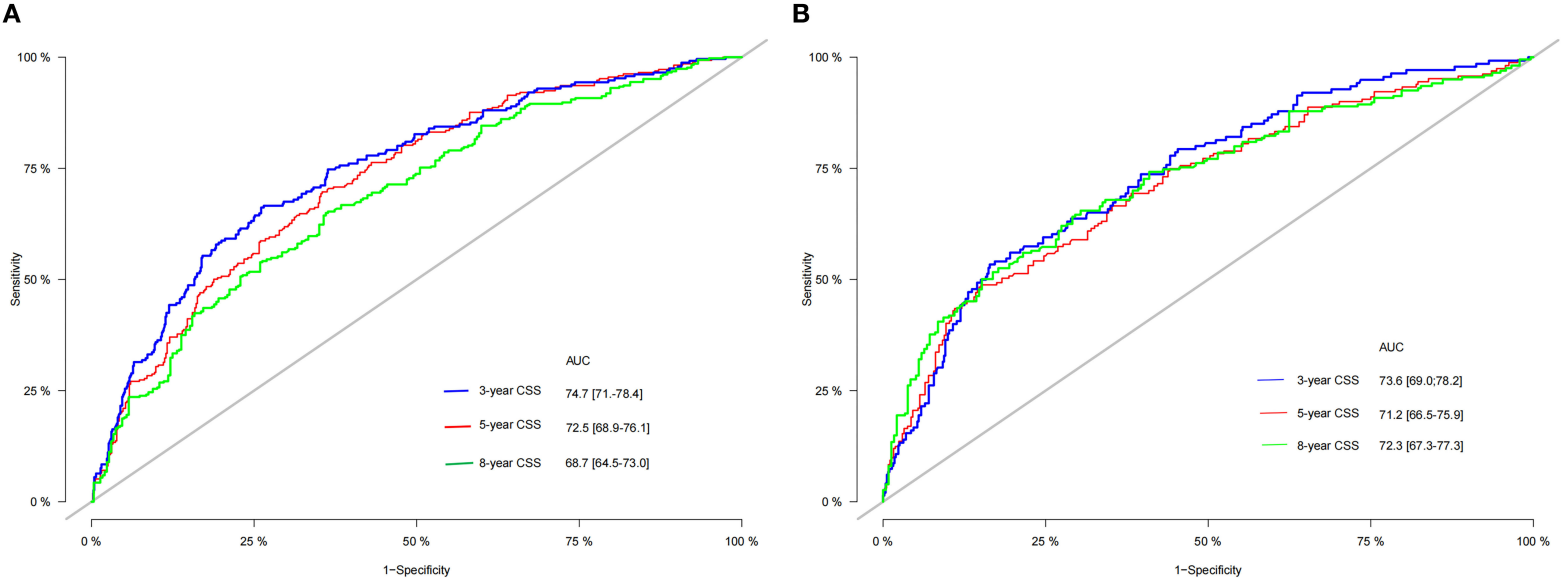
The ROC of 3-, 5-, and 8-year of the training **(A)** and validation **(B)** sets.

The C-index of the training set and the validation set were 0.729 (95% CI 0.702–0.756) and 0.735 (95% CI 0.702–0.768), respectively. This showed that the nomogram has good discrimination. The calibration curves of the training set and the validation set showed that the predicted value of the nomogram is highly consistent with the observed value (Figures 3A–F), which proved that the nomogram has good accuracy. In the training set, the 3-, 5-, 8-year AUC of the nomogram were 74.7 (95% CI 71–78.4), 72.5 (95% CI 68.9–76.1), 68.7 (95% CI 64.5–73.0), respectively. In the validation set, the 3-, 5-, 8-year AUC of the nomogram were 73.6 (95% CI 69.0–78.2), 71.2 (95% CI 66.5–75.9), and 72.3 (95% CI 67.3–77.3), respectively (Figures 4A,B). The AUC results once again proved the accuracy and discrimination of the nomogram. These validations showed that the nomogram was at least 70% accurate, especially for predicting medium-term survival.

### Clinical Application of the Nomogram

**Figure 5:**
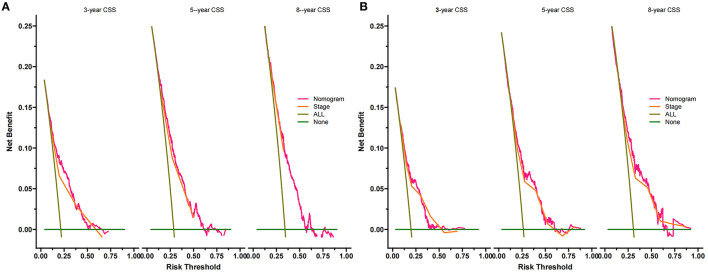
Decision curves of the nomogram predicting CSS in the training set **(A)** and the validation set **(B)**. The x-axis is the threshold probability, and the y-axis is the net benefit. The green line indicates that no patients have died, and the dark green line indicates that all patients have died. When the threshold probability is between 10 and 50%, the net benefit of the model exceeds all deaths or no deaths.

**Figure 6:**
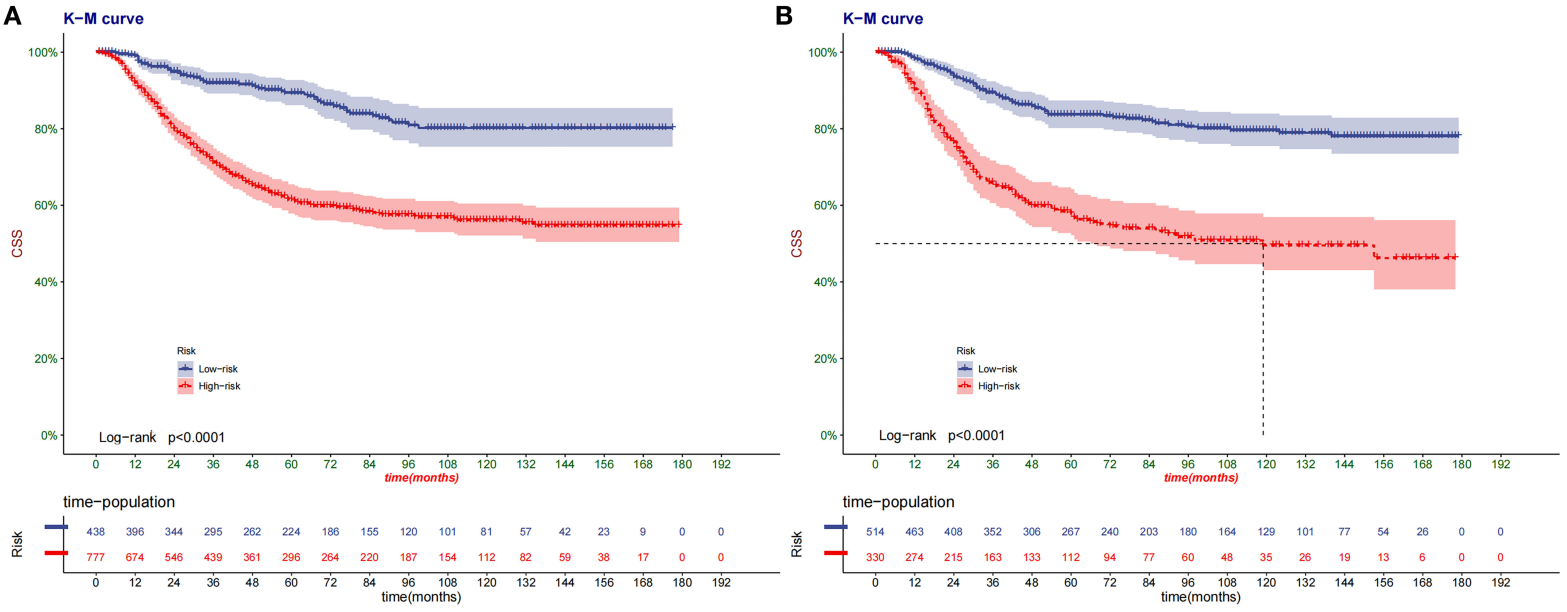
Kaplan–Meier curves of CSS for children in the low- and high-risk groups in the training set **(A)** and validation set **(B)**.

**Figure 7:**
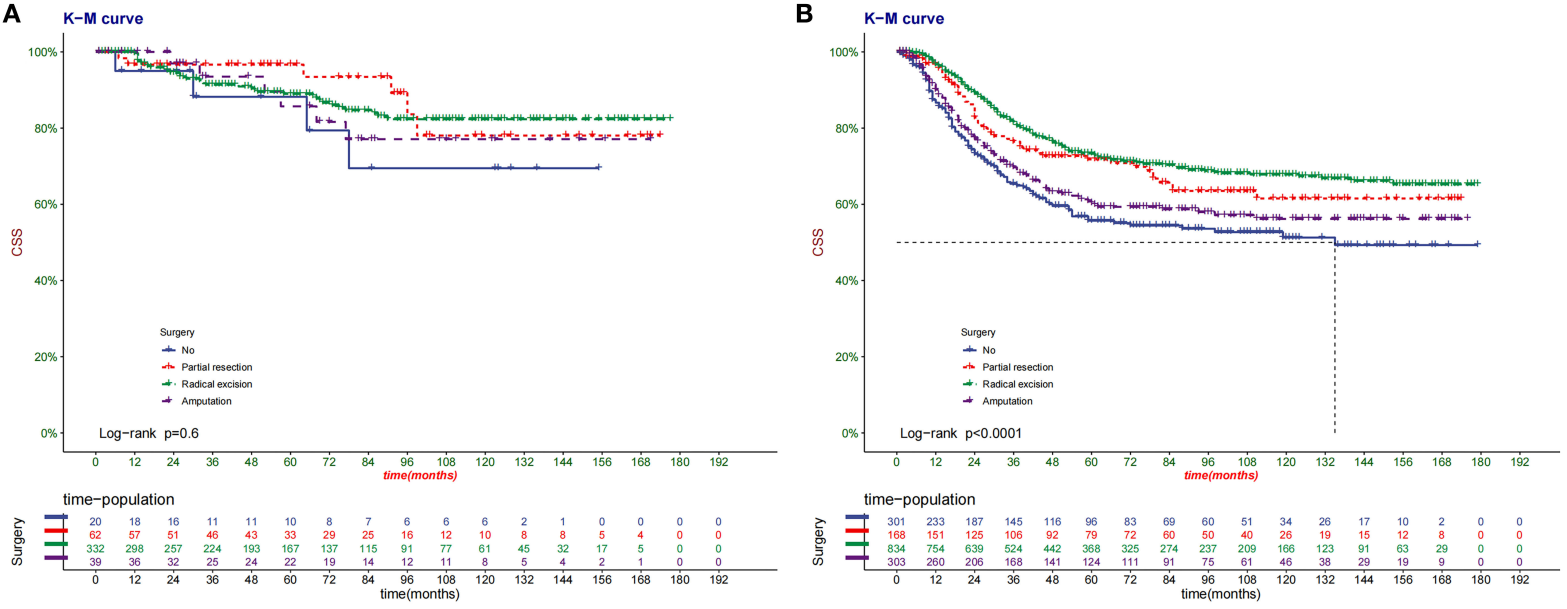
Kaplan–Meier curves of CSS for children with different surgery in the low- **(A)** and high-risk **(B)** groups in total set.

The DCA of the training set and the validation set showed that the clinical value of the nomogram is higher than that of the tumor stage (Figures 5A,B). According to the score of the nomogram, the patients were divided into two groups: low-risk group (total score <75.46) and high-risk group (total score ≥75.46). The Kaplan-Meier curve showed that high-risk patients have lower survival rates than low-risk patients (Figures 6A,B). In the total set, the low-risk group's 3-year, 5-year, and 8-year CSS rates were 92.2, 89.7, and 81.5%, the high-risk group were 75.7, 67.5, and 63.3%, respectively. In addition, we analyzed the impact of surgical methods in different risk groups on the survival probability of patients. In the low-risk group, radical resection was the primary surgical method, and there was no significant difference in the survival rate of various surgical methods (Figure 7A). In the high-risk group, patients with radical resection had the highest survival rate, followed by partial resection, amputation and no surgery (Figure 7B).

## Discussion

OSC is the most common malignant bone tumor in children. The application of neoadjuvant chemotherapy has increased the survival rate of patients with OSC from 15 to 17% of surgical treatment alone to 70% (25). However, the survival rate of patients with OSC has not improved significantly in the past 30 years. As the second most common bone tumor in children, EWS has a lower mortality rate than OSC, but metastatic EWS is still a fundamental cause of harm to children's health (26). Because it is a highly aggressive cancer, it can metastasise to bone marrow, lung and other tissues in the early stage of the disease (27–29): children's malignant bone tumors, especially OSC, hurt patients. Accurately assessing prognostic factors can improve the prognosis and help clinicians effectively evaluate survival and make treatment decisions.

We collected 2,059 children with OSC and EWS from the SEER database. The results showed that the survival rate of children with EWS was significantly higher than that of OSC. More whites than blacks and other races and more boys than girls in all children. The median age of all children was 13 years old, and the peak incidence was 12–15 years old. This study found six independent risk factors through univariate and multivariate Cox regression models, including age, surgery, stage, primary site, tumor size, and histological type. The nomogram includes these factors to establish a predictive model to predict the 3-, 5-, 8-year CSS of childhood OSC and EWS. We first use a nomogram to predict and validate CSS in childhood OSC and EWS. This nomogram has good accuracy and reliability and is of great significance to clinical patients. The nomogram is user-friendly and can be applied to everyone with simple learning.

Previous studies have found prognostic factors for OSC and EWS. However, no study has used these influencing factors to construct a prediction model for survival prediction of children with a bone tumor. Although the influencing factors we identified have previously been shown to be independent risk factors, we integrated these risk factors to construct a nomogram predicting long-term survival in children with OSC and EWS.

Previous studies have shown that various prognostic factors (age, tumor location, type of surgery, local recurrence) will affect the survival of patients (30–32). Our study found that age is an independent risk factor for malignant bone tumors in children, and the survival rate of older children is lower, the same as previous research results (33, 34). Lee et al. (35) found that fewer adult patients receive chemotherapy, which may also be a reason. In addition, we found that the tumor's location near the central axis (spine, pelvis, thorax) has a significantly higher risk of death than the limbs. Like previous studies, axial tumor location predicts the worst prognosis (36, 37). Wan et al. (38) also found that the survival rate of EWS of the spine and pelvis was lower. Because the tumors in the axial position are easier to infiltrate vital organs and metastases far away than the tumors of the limbs. Oberlin et al. (39) believe that bone tumors of the limbs can be removed by surgery, but it is more difficult to remove the axial bone's tumors. Similarly, as in a previous report (40), our study found that tumor size is also a significant risk factor. Large tumors are prone to metastasis, indicating a worse prognosis.

In addition, surgical treatment of bone tumors in children, especially in extremity tumors, is still an important issue. With the widespread development of neoadjuvant chemotherapy and standardized surgery, the survival rate of patients with malignant bone tumors has improved significantly, and limb salvage has become the primary surgical method in treating malignant bone tumors. This treatment model can preserve the function of the limbs and joints and effectively reduce the metastasis and recurrence of OSC (41). Whether it is the low-risk or high-risk group, radical salvage resection has the highest survival rate in our study. However, overall, patients with surgery have a higher survival rate than those without surgery.

Chemotherapy and surgery are still the main treatments for OSC (1). In our study, almost all patients received chemotherapy. In addition, radiotherapy seems to be controversial for treating bone tumors in children. Because surgery is still a radical cure for malignant bone tumors, it will only be considered if cancer cannot be removed entirely (42). For children, radiotherapy may cause developmental delay of bones or organs. Even if it has a therapeutic effect on tumors, it will also seriously affect the quality of life in the future. Our research also found that only a few patients received radiotherapy, similar to previous studies (43).

It is worth mentioning that our analysis found that sex, race, and tumor grade are not independent risk factors. Previous studies have found that they are indeed related to the patient's survival prognosis (44–46). Regarding the grade of the tumor, it may be related to too many cases of unclear grades. Whether race and sex factors affect, the prognosis needs further research.

However, our research still has certain limitations. First, because our study is a retrospective case study based on the SEER database, some possible variables such as surgical margins, tumor recurrence, genetic factors, etc., are not available. However, we have included essential variables such as tumor size, tumor location, pathological type and other vital elements that determine the prognosis and will not cause devastating deviations. Second, we included patients from 2004 to 2018. With the improvement of treatment methods, the survival rate of patients in different years should be different. However, we stratified by the year of diagnosis and found no significant difference, indicating that it did not affect the results. Finally, all the data used in our study were downloaded from the SEER database, and the constructed nomogram lacks external data to validate. Therefore, it is necessary to use external validation further to test the accuracy and reliability of the prediction model. Next, we will conduct further prospective studies in our hospital to externally validate the accuracy of this prediction model further to promote the clinical application.

## Conclusion

We constructed a new nomogram to predict the CSS of children with OSC and EWS. In addition, we found that age, surgery, stage, primary site, tumor size, and histological type are important risk factors affecting children with OSC and EWS. The discovery of risk factors and the Construction of nomograms can help doctors accurately grasp the prognosis of patients, answer patient consultations and help patients make clinical decisions.

## Data Availability Statement

Publicly available datasets were analyzed in this study. This data can be found here: https://seer.Cancer.gov/.

## Author Contributions

JW and CZ designed the study. CZ, JW, JL, ML, LJ, and XT collected and analyzed the data. JW drafted the initial manuscript. CZ, TM, JL, ZZ, and DH revised the article critically. CZ, JL, DH, ML, and XT reviewed and edited the article. All authors approved the final manuscript.

## Funding

This work was funded by Special Key Project of Chongqing Technology Innovation and Application Development (No. Cstc2019jscx-tjsbX0003).

## Conflict of Interest

The authors declare that the research was conducted in the absence of any commercial or financial relationships that could be construed as a potential conflict of interest.

## Publisher's Note

All claims expressed in this article are solely those of the authors and do not necessarily represent those of their affiliated organizations, or those of the publisher, the editors and the reviewers. Any product that may be evaluated in this article, or claim that may be made by its manufacturer, is not guaranteed or endorsed by the publisher.

## Abbreviations

OSC: Osteosarcoma
EWS: Ewing's sarcoma
SEER: the Public Surveillance, Epidemiology, and End Results
CSS: cancer-specific survival
C-index: concordance index
AUC: area under the receiver operating characteristic curve
DCA: decision curve analysis.
